# Dynamic activation of Wnt, Fgf, and Hh signaling during soft palate development

**DOI:** 10.1371/journal.pone.0223879

**Published:** 2019-10-15

**Authors:** Eva Janečková, Jifan Feng, Jingyuan Li, Gabriela Rodriguez, Yang Chai

**Affiliations:** Center for Craniofacial Molecular Biology, University of Southern California, Los Angeles, California, United States of America; University of Colorado Boulder, UNITED STATES

## Abstract

The soft palate is a key component of the oropharyngeal complex that is critical for swallowing, breathing, hearing and speech. However, complete functional restoration in patients with cleft soft palate remains a challenging task. New insights into the molecular signaling network governing the development of soft palate will help to overcome these clinical challenges. In this study, we investigated whether key signaling pathways required for hard palate development are also involved in soft palate development in mice. We described the dynamic expression patterns of signaling molecules from well-known pathways, such as Wnt, Hh, and Fgf, during the development of the soft palate. We found that Wnt signaling is active throughout the development of soft palate myogenic sites, predominantly in cells of cranial neural crest (CNC) origin neighboring the myogenic cells, suggesting that Wnt signaling may play a significant role in CNC-myogenic cell-cell communication during myogenic differentiation in the soft palate. Hh signaling is abundantly active in early palatal epithelium, some myogenic cells, and the CNC-derived cells adjacent to the myogenic cells. Hh signaling gradually diminishes during the later stages of soft palate development, indicating its involvement mainly in early embryonic soft palate development. Fgf signaling is expressed most prominently in CNC-derived cells in the myogenic sites and persists until later stages of embryonic soft palate development. Collectively, our results highlight a network of Wnt, Hh, and Fgf signaling that may be involved in the development of the soft palate, particularly soft palate myogenesis. These findings provide a foundation for future studies on the functional significance of these signaling pathways individually and collectively in regulating soft palate development.

## Introduction

The vital functions of the craniofacial region are facilitated by a complex system of “tubes” and “cavities” [1]. Two major cavities of the craniofacial region are divided by the palate, which serves as the floor of the nasal cavity as well as the roof of the oral cavity. The palate itself is a heterogeneous structure with complex developmental origins. The primary palate is formed by the posterior expansion of the frontonasal process, whereas the secondary palate is formed by the fusion of paired palatal shelves [2–4]. The secondary palate can be further divided into the hard palate (the palatine process of the maxilla and the palatine bone) and soft palate (consisting of muscles). The soft palate is the more posterior portion of the secondary palate and forms part of a bigger functional system, the oropharyngeal complex, which functions in swallowing, speech, breathing, and hearing [5]. These functions are affected by soft palate clefting and have a life-long impact on the health, social integration and overall quality of life of these patients [6, 7]. Although isolated cleft of the soft palate is considered a mild form of cleft palate, restoring the proper functions of the soft palate is a very challenging task for surgeons because the soft palate muscle fibers in such cases are few in number, disoriented and low in regenerative capacity, and their function may be compromised by fibrosis [8, 9]. In light of the disrupted crucial functions that arise from soft palate clefts, understanding the molecular signaling network that controls soft palate development is critical for addressing longstanding challenges in the clinical treatment of cleft soft palate.

The soft palate consists of five muscles in humans and four in mice: the tensor veli palatini (TVP), levator veli palatini (LVP), palatoglossus (PLG) and palatopharyngeus (PLP) are present in both species, whereas the musculus uvulae is only found in humans [10]. At the cellular level, the soft palate region is comprised of CNC-derived cells, cranial paraxial mesoderm and pharyngeal ectoderm [10, 11]. Close interaction between CNC-derived mesenchyme and myogenic cells derived from cranial paraxial mesoderm is required during the development of craniofacial muscles, as signals from CNC-derived mesenchyme guide myogenic progenitors into the soft palate region and instruct myogenic cells to differentiate [10].

Multiple signaling pathways, for example Wnt, Tgf-β, Hh and Fgf, have been shown to regulate palatogenesis [4, 12–14]. Wnt signaling is crucial for regulating craniofacial development; loss or gain of Wnt signaling function can cause severe craniofacial malformations, including cleft palate, indicating that precisely regulated Wnt signaling is a prerequisite for normal craniofacial morphogenesis [15–19]. In particular, Wnt3 and Wnt9b are associated with orofacial clefts in both humans and mice [20–22]. Wnt signaling also plays a role during muscle development, including tongue development [14, 23, 24]. Canonical Wnt signaling is a necessary regulator of progenitor cells and myofibers during early fetal development, myoblast differentiation and myoblast migration in the tongue muscles [24, 25]. Recently, canonical Wnt signaling was also identified to be crucial in the final stage of myogenesis, myoblast fusion, through regulating nephrin [23]. In soft palate development, Wnt signaling is downregulated after conditional deletion of Tgf-β signaling from the palatal epithelium, which is associated with soft palatal muscle defects [12]. Hh signaling is critical for early embryonic development and plays a key role in patterning, survival and proliferation of early CNC and other cell populations [26, 27] and is involved in the development of multiple craniofacial tissues [27–29]. During hard palate development, Hh signaling establishes the oro-nasal and antero-posterior axes through epithelial-mesenchymal interactions [30–33]. Hh signaling also plays a role during muscle development, including that of the tongue, and is crucial for post-migratory CNC proliferation in the craniofacial region [14, 27]. Both loss- and gain-of-function mutations in Fgf receptors and ligands are associated with numerous congenital disorders affecting the craniofacial region, including non-syndromic and syndromic cleft palate [34–39]. Syndromes associated with cleft palate include those arising from mutations in *FGFR1-3*; e.g. Apert syndrome (*FGFR2*), Muenke syndrome (*FGFR3*), Crouzon syndrome (*FGFR2*, *FGFR3*) and Hartsfield syndrome (*FGFR1*) [39]. The role of Fgf signaling has also been shown in skeletal muscle development, especially in repressing the differentiation of myoblasts [40]. Together with Wnt signaling, Fgf signaling is essential for specification of paraxial mesoderm during early fetal myogenesis [41]. Recently, Fgf signaling was shown to play a role in Dlx5-mediated development of the soft palate muscles [13]. There is a close interaction among the Wnt, Hh and Fgf signaling pathways during the development of the hard palate [4, 42]. Wnt signaling acts upstream of Hh during hard palate development [14, 43]. Hh cooperates with Fgf signaling via epithelial-mesenchymal interactions, especially through Fgfr2/Fgf10, during the development of the hard palatal shelves [31]. Fgf signaling is also modulated by canonical Wnt signaling during early craniofacial development [18] and there is a regulatory feedback loop between Wnt11 and Fgfr1 during hard palate development [44].

Since most previous molecular studies have focused on the hard palate, the regulatory mechanism of soft palate development has just started to be unveiled. In order to investigate and characterize the signaling network that controls the interactions between CNC-derived mesenchyme, mesoderm-derived myogenic cells and pharyngeal ectoderm-derived epithelium, our current study focuses on the expression of active Wnt, Hh and Fgf signaling during soft palate development in mice. We have analyzed the expression and activation patterns of Wnt, Hh and Fgf pathway members throughout the soft palate region in cells of ectodermal, CNC and mesodermal origin during embryonic development and revealed their dynamic involvement in soft palate muscle development. This study identifies dynamic signaling expression patterns during the development of the soft palate and lays the groundwork for improved treatment of clefts of the soft palate.

## Material and methods

### Animals and procedures

Animal studies were completed in accordance with federal regulations and approval from the Institutional Animal Care and Use Committee (IACUC) at the University of Southern California (Protocol Number: 9320). C57BL/6J and reporter mouse lines *Gli1-LacZ*^*+/-*^ [JAX#008211, [45]] and *Axin2-LacZ*^*+/-*^ [JAX#009120, [46]] were used for this study. Animals were euthanized by carbon dioxide overdose followed by cervical dislocation. Embryos collected on embryonic day (E)14.5, E16.5 or E18.5 were fixed overnight in 10% neutral buffered formalin solution (Sigma, HT501128), decalcified in ethylenediaminetetraacetic acid (EDTA, pH 7.1–7.3, Alfa Aesar, A15161) for 1 week and processed for cryosectioning or paraffin embedding and sectioning (8 μm). Briefly, for the paraffin sectioning protocol, samples were washed in DEPC-PBS three times for 5 minutes, dehydrated through an increasing ethanol series (4 hours each of 30%, 50%, 70%, 80%, and 95% EtOH followed by 2 x 4 hours in 100% EtOH), then treated with xylene (for 3 hours or until clear), xylene/paraffin for 45 minutes, and finally paraffin (six 1-hour baths, one bath overnight). For cryosectioning, samples were washed in DEPC-PBS three times for 5 minutes each, dehydrated in 15% sucrose solution (Sigma, S0389), diluted in DEPC-PBS and 30% sucrose/50% OCT solution, and embedded in Tissue-Tek^®^ OCT Compound (Sakura, 4583). In the case of *LacZ*^*+/-*^ samples, 0.2% glutaraldehyde with 2mM MgCl_2_ was used for fixation overnight, followed by decalcification in EDTA (pH 7.1–7.3) with 2mM MgCl_2_ for one week. Samples were dehydrated in sucrose solutions with 2mM MgCl_2_ as described above and embedded in OCT.

### Histological and X-gal staining

Hematoxylin and eosin staining was performed on deparaffinized slides according to standard procedure. Sections from *LacZ* reporter mice were subjected to X-gal staining. After rinsing the slides with PBS and 2mM MgCl_2_ to remove the OCT, they were placed in X-gal staining solution (1M MgCl_2_, 1% NaDOC, 1% NP-40, 50 mM K Ferri, 50 mM K Ferro, 1 M Tris (pH 7.3), 40 mg/ml X-gal, 1x PBS) at 37°C overnight or over two nights, if necessary. After staining, slides were washed twice with PBS for 5 minutes each, fixed in formalin for 30 minutes, counterstained with Nuclear Fast Red for 3 minutes, and washed with distilled water and 70% EtOH twice for 2 minutes each. Slides were stained in eosin for 75 seconds, then subjected to two 95% EtOH rinses and two 2-minute washes in 100% EtOH before final clearance in xylene (two times 2 minutes) and mounted.

### RNAscope and immunohistochemistry

Sections were air dried for 15 minutes at room temperature and 15 minutes at 37°C. OCT was removed from the slides with a 5-minute wash in distilled water and slides were placed into pre-heated target retrieval reagent (Advanced Cell Diagnostics, ACD, 322000) for 15 minutes at 99°C. For *in situ* hybridization, RNAscope^®^ 2.5 HD detection reagent–red kit was used (ACD, 322360) according to the manufacturer’s instructions. The probes used for this study were Mm-Axin2 (400331), Mm-Etv4 (458121), Mm-Etv5 (316961), Mm-Fgfr1 (443491), Mm-Fgfr2 (443501), Mm-Fgfr3 (440771), Mm-Fgfr4 (443511), and Mm-Gli1 (311001). After detection of the mRNA signal, slides were fixed in formalin for 10 minutes, and washed in PBS. Immediately after that, pre-heated citrate-based antigen unmasking solution (Vector, H-3300) was applied for 15 minutes at 99°C. After washes in 0.1% Tween20/PBS (PBST), sections were incubated with blocking reagent (Perkin Elmer, FP1012) for 1 hour followed by primary antibody (myosin heavy chain, MHC, Developmental Studies Hybridoma Bank: MF20) diluted 1:10 overnight at 4°C. The second day, after three five-minute washes in PBS, secondary antibody from the M.O.M. kit (Vector, MP-2400) was applied for 10 minutes followed by detection of the signal using peroxidase substrate kit DAB (Vector, SK-4105). After signal development, slides were counterstained with haematoxylin, fixed for 10 minutes in formalin, and mounted with aqueous mounting medium (Vector, H-5501). For MHC immunofluorescence, after incubation of the primary antibody overnight, secondary antibody (Alexa Fluor 488 anti-mouse, 1:200, Invitrogen, A11001) was applied for two hours at room temperature, followed by three washes in PBST and 5 minutes counterstaining with DAPI (Invitrogen, D1306). For immunofluorescence staining of active β-catenin and its co-localization with MHC, samples were treated with 3% hydrogen peroxide solution, pre-heated in citrate-based antigen unmasking solution (Vector, H-3300, 15 minutes at 99°C) and treated with blocking reagent (Invitrogen, B40922) for 1 hour at room temperature. After incubation of the primary antibody overnight (myosin heavy chain, MHC, Developmental Studies Hybridoma Bank: MF20, 1:10 and active beta-catenin, Cell Signaling: S45, D2UBY, 1:100), secondary antibody (Alexa Fluor 568 anti-mouse, 1:200, Invitrogen, A11004 and anti-rabbit IgG poly HRP conjugate) was applied for two hours at room temperature, followed by three washes in PBST, tyramide development for 3 minutes (Alexa Fluor 488 Tyramide SuperBoost, Invitrogen, B40922) and counterstaining with DAPI (Invitrogen, D1306).

## Results

### Activation of Wnt signaling during soft palate development

**Fig 1:**
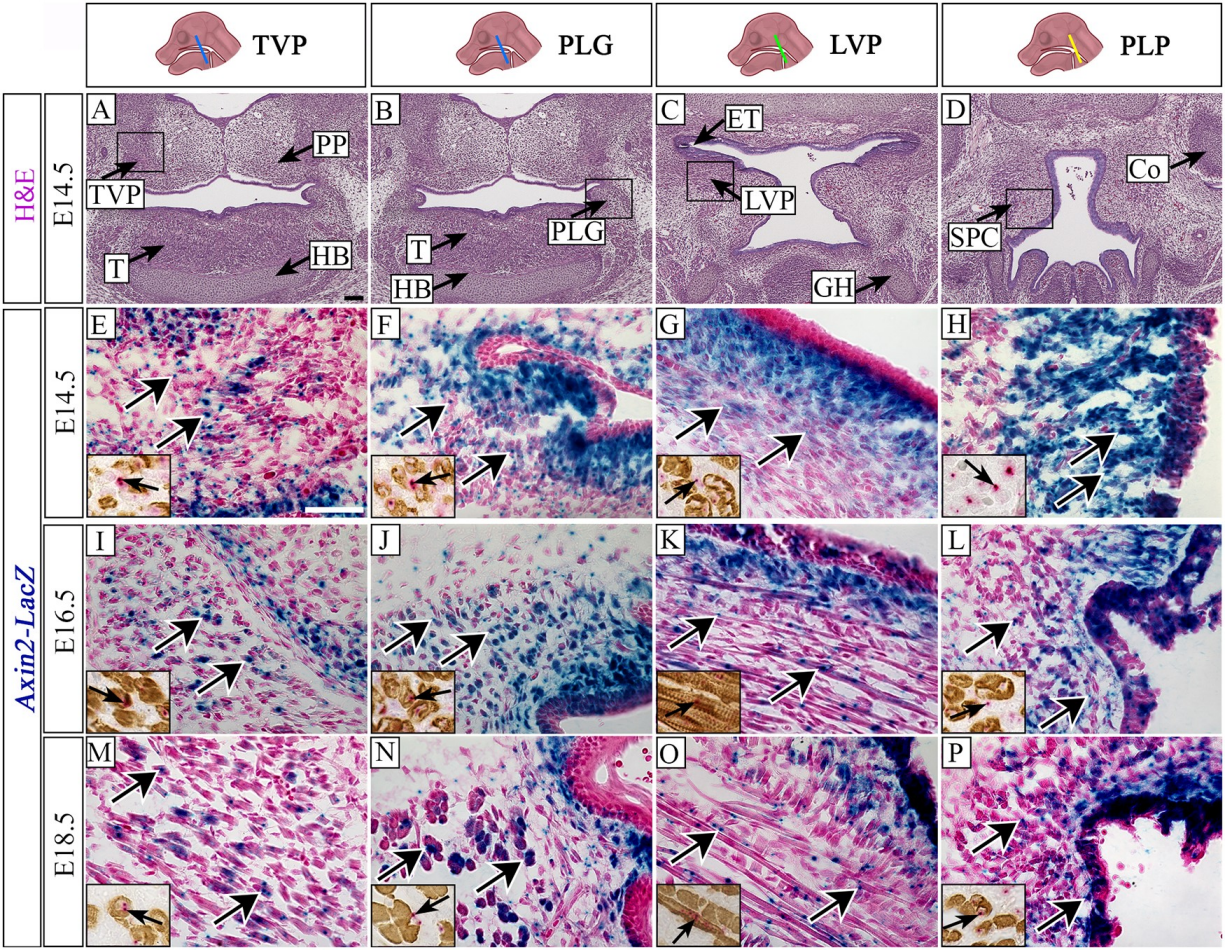
*Axin2* is expressed throughout the embryonic development of the murine soft palate region. (A-D) Hematoxylin and eosin staining of frontal sections of a mouse head at E14.5. Schematic drawings of the mouse head in the top panel depict the position and angle of the sections. Boxed areas in A-D respectively indicate approximate locations of higher magnification images (E-P) of TVP (E,I,M), PLG (F,J,N), LVP (G,K,O) and PLP (H,L,P). (E-P) Visualization of *Axin2-LacZ* reporter mice at E14.5 (E-H), E16.5 (I-L) and E18.5 (M-P). Insets in E-P show expression of *Axin2* mRNA detected by RNAscope (pink) and MHC protein in comparable regions to E-P visualized by immunohistochemistry (brown). Arrows indicate *Axin2*-positive cells. Co, cochlea; ET, Eustachian tube; GH, greater horns of the hyoid bone; HB, hyoid bone; H&E, hematoxylin and eosin staining; LVP, levator veli palatini; PLG, palatoglossus; PLP, palatopharyngeus; PP, pterygoid plate; SPC, superior pharyngeal constrictor; T, tongue; TVP tensor veli palatini. Scale bar in A = 100 μm (A-D), scale bar in E = 50 μm (E-P), 27 μm (insets in E-P).

As previously described, in mice, the TVP and PLG are the most anterior soft palate muscles [9, 10], and can be seen at E14.5 in coronal section with the tongue (T), hyoid bone (HB) and pterygoid plate (PP) (Fig 1A and 1B). The LVP is located posterior to the TVP, where the greater horns of the hyoid bone (GH) can be observed in histological sections, together with the opening of the Eustachian tube (ET) (Fig 1C). The PLP is located in the most posterior part of the soft palate at the level of the cochlea (Co) and superior pharyngeal constrictor (SPC), but it is not yet differentiated at E14.5 (Fig 1D).

Previous studies have demonstrated that Wnt signaling, particularly canonical Wnt signaling, is involved during the development of the palate and during muscle development [14, 16, 20, 23–25, 47, 48]. Therefore, we hypothesized that Wnt signaling may also be active during soft palate myogenesis and evaluated the expression of *Axin2*, which plays an important role in the regulation of β-catenin and can be used as a readout of active canonical Wnt signaling, by analyzing *Axin2-LacZ* reporter mice and by analyzing *Axin2* mRNA expression. LacZ staining of *Axin2-LacZ* mice indicated *Axin2* expression as early as E14.5 in the whole soft palate region. In the TVP region at E14.5, *Axin2* expression was predominantly present in CNC-derived mesenchymal cells and in a few MHC+ muscle fibers (Fig 1E). CNC-derived mesenchymal cells also showed abundant expression of *Axin2* in the region surrounding the PLG, with the highest expression in the region close to the epithelium (Fig 1F). A few *Axin2*+ myogenic cells were also found in the PLG region (Fig 1F). More posteriorly, *Axin2* expression was detectable predominantly in the CNC-derived mesenchymal cells surrounding initial LVP muscle fibers (Fig 1G). At E14.5, although the muscle fibers of PLP were not yet differentiated, extensive expression of *Axin2* was detectable in the putative region of the PLP, mainly consisting of CNC-derived mesenchymal cells (Fig 1H). Later in development, muscles of the soft palate increase in size and number [10]. In the TVP and PLG region at E16.5, *Axin2* expression was detectable primarily in the CNC-derived mesenchymal cells (Fig 1I and 1J). In the region of the LVP and PLP, the expression pattern was similar: *Axin2* was widely expressed in the CNC-derived mesenchymal cells surrounding the myogenic cells, and a few MHC+ cells were also *Axin2*+ (Fig 1K and 1L). At E18.5, *Axin2* expression persisted in CNC-derived mesenchymal cells and in the MHC+ myogenic fibers of individual soft palatal muscles at the level of the TVP (Fig 1M), PLG (Fig 1N), LVP (Fig 1O) and PLP (Fig 1P). At all examined stages, expression of *Axin2* was also observed in the palatal epithelium (Fig 1F–1H, 1J–1L and 1N–1P). These results were confirmed also by assessing the active β-catenin signaling (S1 Fig). β-catenin is the mediator of canonical Wnt signaling, transducing the signal upon specific Wnt ligand and receptor binding, mediating cellular response. The detection of active β-catenin therefore indicates activation of canonical Wnt signaling. Similar to Axin2 expression, activated β-catenin was predominantly present in the CNC-derived mesenchymal cells and less expression was seen in the myogenic cells (S1 Fig).

These results demonstrate the broad and persistent expression of Wnt signaling in the course of embryonic soft palate development, predominantly in the CNC-derived mesenchymal cells surrounding the myogenic fibers, but also in the MHC+ and epithelial cells of the soft palate region. Consistent with previous studies, our analysis suggests that Wnt signaling is likely to be significant for soft palate muscle establishment, differentiation, and fusion.

### Hh signaling activity during soft palate development

**Fig 2:**
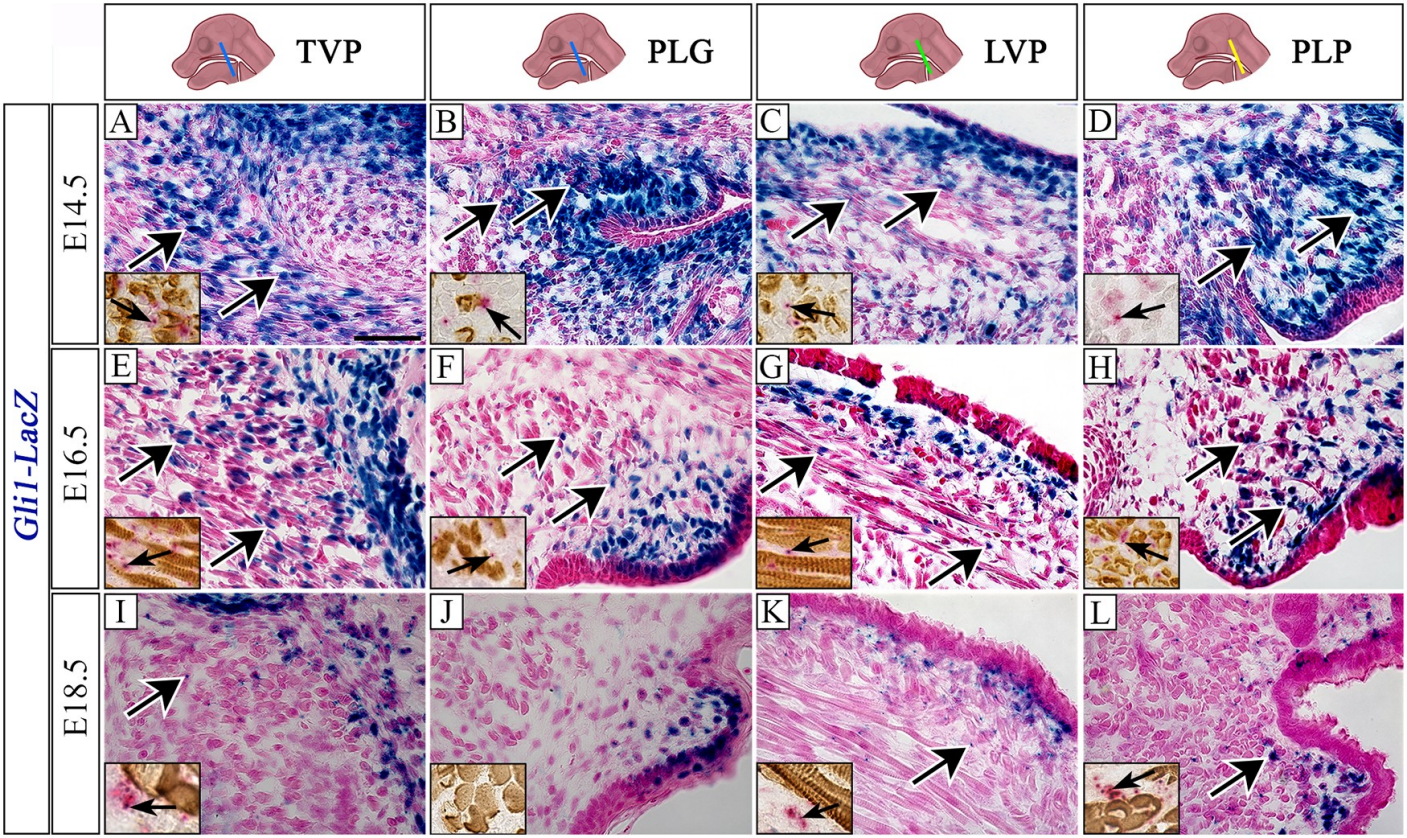
*Gli1* is dynamically expressed in the soft palate region. (A-L) Visualization of *Gli1-LacZ* reporter mice at E14.5 (A-D), E16.5 (E-H), and E18.5 (I-L). Insets in A-L show expression of *Gli1* by RNAscope (pink) and MHC immunohistochemistry (brown) in comparable regions to A-L. Arrows indicate *Gli1* positive cells. Schematic drawings of the mouse head in the top panel depict the position and angle of the sections. LVP, levator veli palatini; PLG, palatoglossus; PLP. palatopharyngeus; TVP, tensor veli palatini. Scale bars = 50 μm (A-L), 27 μm (A-L).

Hh signaling is involved in early embryonic development and plays an indispensable role in reciprocal epithelial-mesenchymal interactions guiding palatal outgrowth and tongue muscle development [14, 27, 32, 33]. To test our hypothesis that Hh signaling may be involved in soft palate muscle development, we analyzed the expression of *Gli1*, a transcription factor activated by Hh signaling, via *Gli1-LacZ* mice and *Gli1* mRNA expression. At E14.5, *Gli1* expression was plentiful at the level of the TVP in the CNC-derived mesenchymal cells surrounding myogenic cells, and a few myogenic MHC+ cells were also *Gli1+* (Fig 2A). In the PLG region, *Gli1* expression was also abundant in the CNC-derived mesenchymal cells, and only a few *Gli1*+ MHC+ myogenic cells were visible (Fig 2B). More posteriorly, at the level of the LVP, where the first myogenic fibers of LVP could be observed, Gli1 signal was abundant in the CNC-derived mesenchyme surrounding the myogenic fibers close to the epithelium, whereas almost no *Gli1*+MHC+ myogenic cells were detectable (Fig 2C). In the putative region of the PLP primordia, abundant expression of *Gli1* was present in the CNC-derived mesenchymal cells (Fig 2D). Later in development, at E16.5, overall fewer *Gli1*+ cells were observed in the soft palatal region. The majority of the *Gli1*+ cells persisted in the CNC-derived mesenchyme both surrounding the myogenic fibers and adjacent to the epithelium (Fig 2E–2H). At E18.5, overall Gli1 expression remained low, except a few *Gli1*+ cells were detected at the level of the TVP in the CNC-derived mesenchyme (Fig 2I). The PLG region showed scattered *Gli1* expression in CNC-derived mesenchyme surrounding the muscle cells (Fig 2J). The LVP region was nearly absent of *Gli1*+ cells, with only few *Gli1*+ cells present in the CNC-derived mesenchyme close to the epithelium (Fig 2K). In the PLP region, scattered *Gli1* expression persisted in the CNC-derived mesenchymal cells close to the epithelium (Fig 2L). *Gli1* expression was also observed in the epithelium of all the soft palate regions throughout development (Fig 2B–2D, 2F–2H and 2J–2L). This dynamic spatiotemporal expression pattern of *Gli1* suggests the requirement of Hh signaling pathway in the early stages of soft palate development, predominantly in CNC-derived mesenchymal cells, to regulate myogenesis through cell-cell interaction.

### Expression of Fgf signaling during soft palate development

The role of Fgf signaling has been shown in both palatal clefting and skeletal muscle development, especially in repressing the differentiation of myoblasts [34, 39, 40]. We therefore hypothesized that Fgf signaling might play a role in the development of soft palate muscles. We analyzed the expression of *Etv5* and *Etv4*, downstream targets of Fgf signaling, and Fgf receptors (*Fgfr1-4*) to assess whether Fgf signaling is activated during soft palate development.

**Fig 3:**
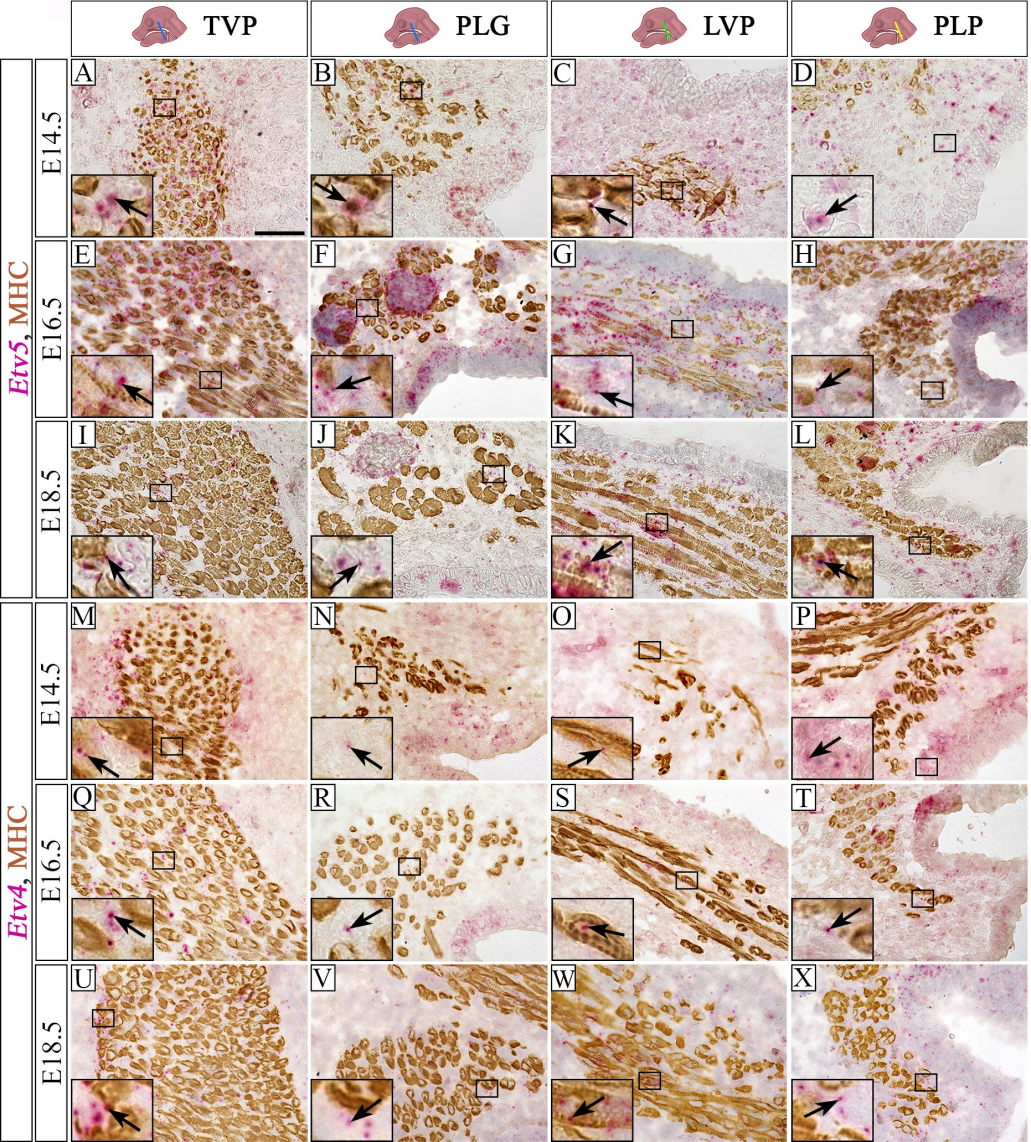
*Etv5* and *Etv4*, downstream targets of Fgf signaling, are abundantly expressed throughout embryonic soft palate muscle development. (A-L) *Etv5* and (M-X) *Etv4* RNAscope (pink) and MHC immunohistochemistry (brown) in the soft palate region of C57BL/6J mice at E14.5 (A-D,M-P), E16.5 (E-H,Q-T), and E18.5 (I-L,U-X). Insets (A-X) show magnified views of *Etv5* (A-L) and *Etv4* (M-X) signal detected by RNAscope (pink) and MHC immunohistochemistry (brown). Arrows indicate *Etv5* and *Etv4* positive cells. Schematic drawings of the mouse head in the top panel depict the position and angle of the sections. LVP, levator veli palatini; MHC; myosin heavy chain; PLG, palatoglossus; PLP, palatopharyngeus; TVP, tensor veli palatini. Scale bars = 50 μm (A-X), 12 μm (insets in A-X).

Early in embryonic soft palate development at E14.5, *Etv5* and *Etv4* expression could be observed predominantly in the CNC-derived mesenchymal cells closely interacting with muscle fibers of the TVP (Fig 3A and 3M) and PLG (Fig 3B and 3N). Few MHC+ myogenic cells of the TVP and PLG expressed *Etv5* (Fig 3A, 3B, 3M and 3N). Initial muscle fibers of the LVP were surrounded by active Fgf signaling in the CNC-derived mesenchymal cells (Fig 3C and 3O). The putative region of the PLP, where only CNC-derived mesenchymal cells could be observed at E14.5, also showed activation of Fgf signaling (Fig 3D and 3P). Later in development, at E16.5 and E18.5, Fgf signaling remained active predominantly in the CNC-derived mesenchymal cells with rare *Etv5* expression in MHC+ cells (Fig 3E–3L and 3Q–3X). Fgf signaling activity was also detected in the epithelium throughout the embryonic development of the soft palate (Fig 3B–3D, 3F–3H, 3J–3L, 3N, 3P, 3R, 3S and 3V–3X).

**Fig 4:**
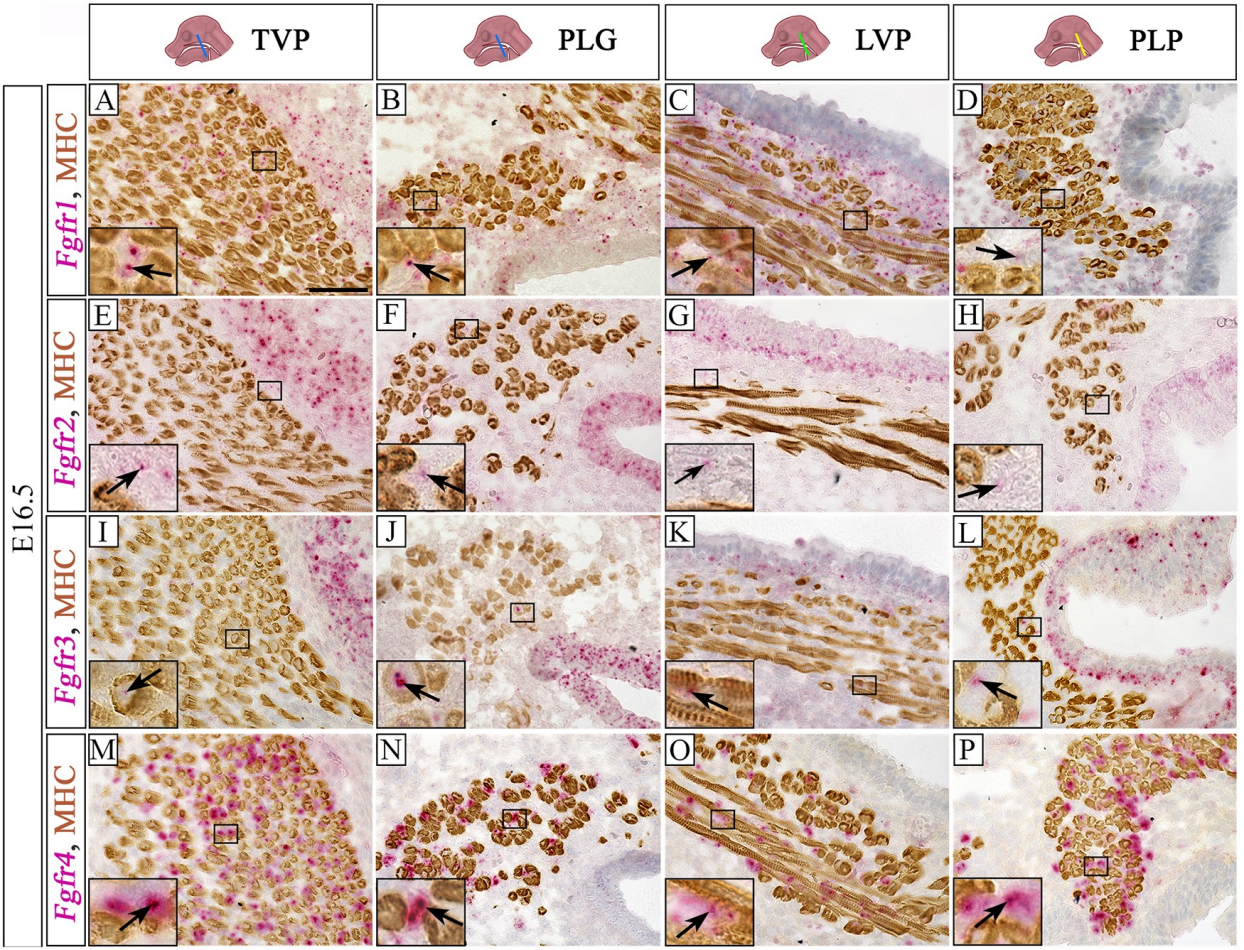
Expression of *Fgfr1*, *Fgfr2*, *Fgfr3*, and *Fgfr4* in the soft palate myogenic sites. (A-P) Co-staining of MHC immunohistochemistry (brown) and Fgf receptor RNAscope (pink) in the soft palate myogenic sites of C57BL/6J mice at E16.5: *Fgfr1* (A-D), *Fgfr2* (E-H), *Fgfr3* (I-L), *Fgfr4* (M-P). Arrows indicate positive signal. Insets (A-P) show magnified views of *Fgfr1* (A-D), *Fgfr2* (E-H), *Fgfr3* (I-L) and *Fgfr4* (M-P) signal detected by RNAscope (pink) and MHC immunohistochemistry (brown). Schematic drawings of the mouse head in the top panel depict the position and angle of the sections. LVP, levator veli palatini; MHC; myosin heavy chain; PLG, palatoglossus; PLP, palatopharyngeus; TVP, tensor veli palatini. Scale bars = 50 μm (A-P), 13.5 μm (insets in A-P).

We further analyzed Fgf receptor activity at E16.5. The expression of *Fgfr1* was predominantly present in the CNC-derived mesenchymal cells and in the epithelium (Fig 4A–4D). Few scattered *Fgfr2* positive cells were in the CNC-derived mesenchyme and epithelium (Fig 4E–4H). Expression of *Fgfr3* was detected in few CNC-derived mesenchymal cells and epithelium (Fig 4I–4L). Among the four Fgf receptors we assessed, *Fgfr4* appeared to be the most abundantly expressed during soft palate development. The majority of the *Fgfr4* signal was in the CNC-derived mesenchymal cells surrounding the muscle fibers at the level of the TVP (Fig 4M), PLG (Fig 4N), LVP (Fig 4O) and PLP (Fig 4P) at E16.5; little expression of *Fgfr4* was detectable in the myogenic fibers. Interestingly, the expression of Fgfr4 was not detected in the palatal shelf epithelium (Fig 4N–4P). Our results describe the spatiotemporal activation pattern of Fgf signaling in soft palate development and suggest its role in this process.

**Fig 5:**
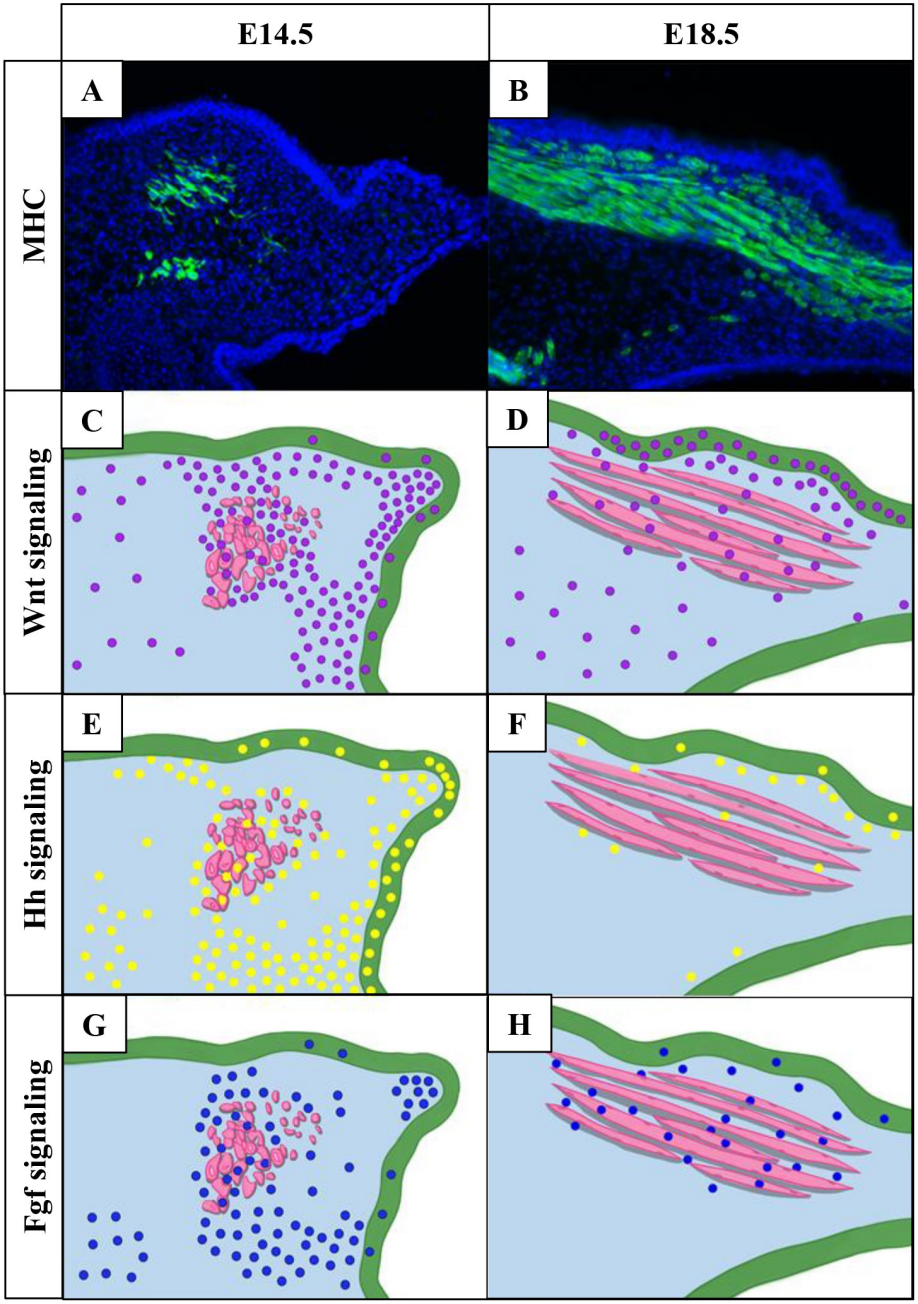
Schematic drawing summarizing the expression of Wnt, Hh, and Fgf signaling in the LVP region. (A-B) Visualization of myogenic fibers by MHC immunofluorescence at E14.5 (A) and E18.5 (B). (C-D) Schematic drawings summarizing Wnt signaling activity (purple dots) at E14.5 (C) and E18.5 (D); (E-F) Hh signaling activity (yellow dots) at E14.5 (E) and E18.5 (F); and (G-H) Fgf signaling activity (dark blue dots) at E14.5 (G) and E18.5 (H). Light blue indicates the CNC-derived mesenchymal cells and green indicates soft palatal epithelium. MHC, myosin heavy chain.

It is particularly important to understand the molecular signaling that controls the development of the LVP, which is the major muscle of the soft palate and performs important functions as a part of the oropharyngeal complex [5]. For this reason, we highlight the LVP in summarizing the molecular signaling network that regulates soft palate muscle development (Fig 5A and 5B). We found that Wnt signaling was abundant in all cell types of the soft palate, especially in the CNC-derived mesenchyme, at E14.5 (Fig 5C) and persisted until E18.5 (Fig 5D). Hh signaling was active mainly in the CNC-derived mesenchymal cells, as well as in a few myogenic and epithelial cells at E14.5 (Fig 5E) and diminished at E18.5 (Fig 5F). Fgf signaling was widespread in the CNC-derived mesenchymal cells but scattered in the myogenic cells and palatal epithelium at E14.5 (Fig 5G) and persisted in the soft palate region at E18.5 (Fig 5H). Taken together, our studies suggest a dynamic signaling network of Wnt, Hh, and Fgf pathways that mediate the interactions between CNC-derived mesenchymal and myogenic cells and/or interactions between epithelium and mesenchyme during soft palate development.

## Discussion

In this study, we show for the first time the detailed spatiotemporal expression patterns of Wnt, Hh and Fgf signaling pathways during embryonic soft palate muscle development with a focus on potential interactions among cells derived from CNC, paraxial mesoderm and pharyngeal ectoderm. We highlight the importance of these signaling pathways during soft palate formation and suggest that they may instruct cell-cell interactions to guide embryonic soft palate muscle development.

### Expression patterns of Wnt, Hh, and Fgf signaling pathways suggest their role in regulating soft palate development

Wnt, Hh, and Fgf are multifunctional signaling pathways and their specificity in regulating organogenesis is achieved through different combinations of their specialized activities, interactions with other signaling molecules/pathways and the specific cellular environments where they reside [49, 50]. The Wnt, Hh and Fgf signaling networks are highly conserved across species, as well as across the development of various organs. For instance, the interplay of these signaling pathways has been shown to regulate the development of scales in zebrafish [51]. In mice, the interplay of this signaling network regulates the growth/patterning of teeth [52], limbs [50], salivary glands [53, 54] and airway smooth muscle [55], as well as the early development of the face [18] and the thalamus [49]. These signaling pathways are also co-expressed during the development of the hard palate [14, 33, 39, 56].

Conventional or conditional knockout of Wnt, Hh, or Fgf signaling in the CNC cells, palatal mesenchyme or epithelium causes severe craniofacial phenotypes, most of which include cleft palate [16, 18, 19, 27, 28, 31, 36, 48, 57–59]. Furthermore, during hard palate development, Wnt signaling acts upstream of both Fgf and Hh signaling [14, 57], and Hh is downstream of Fgf signaling [31]. The co-expression of Wnt, Hh and Fgf signaling pathways we observed in our study might imply their interaction and shared function in embryonic development of the soft palate, especially at early stages, since Hh signaling diminishes in later stages of soft palate development.

Despite the fact that roles for Wnt, Hh and Fgf signaling are also well established in the development of the hard palate, the involvement of these signaling pathways in the soft palate development has not previously been described. Revealing the interaction of the signaling pathways regulating soft palate myogenic development is critical for understanding this process since the complexity of signaling pathways involved reflects the high incidence of cleft palate. It will be crucial to understand how these signaling pathways achieve their specificity in regulating soft palate development. This information will guide us in potential tissue engineering approaches to improve soft palate repair and regeneration.

### Cell-cell interactions in soft palate development

Cell-cell and tissue-tissue interactions are fundamental for the development and function of various organs. Epithelial-mesenchymal interactions are some of the best-studied inductions and are well characterized in the development of numerous organs, such as the tooth [60], lung, kidney [61], mammary gland [62] and many others, including the palate [12, 31]. In the craniofacial region, interactions between CNC cells and mesodermal/myogenic cells are of special interest [4, 11]. The cooperation of different cell types through signaling molecules has been previously suggested for regulation of soft palate development as well [10, 13, 63]. Our data are in agreement with these suggestions and indicate that Wnt, Hh and Fgf signaling are active in all three components of soft palate: CNC-derived mesenchymal cells, myogenic cells, and epithelium. The activity of all these signaling pathways is predominant in the CNC-derived mesenchymal cells during early soft palate development, when the myogenic cells of the soft palate are not differentiated yet (e.g., in the presumptive region of PLP at E14.5), and Wnt and Fgf signaling activity persists in the CNC-derived mesenchymal cells until the later stages of soft palate development. Previously published results revealed that Wnt signaling mediates cell-cell interactions of fibroblasts with myocytes and other cell populations after cardiac injury [64]. Furthermore, defective soft palate muscle formation is observed subsequent to decreased Wnt signaling in CNC-derived cells [12]. Hh and Fgf signaling play important roles in cells of CNC origin, interacting with tongue myogenic progenitor cells [27, 65]. In hard palate development, epithelial Hh signaling closely interacts with Fgf signaling molecules in the mesenchyme, specifically Fgf10 and Fgfr2 [31]. Similarly, downregulation of Fgf signaling in Dlx5+ cells disrupts tissue-tissue interactions, leading to aberrant development of the muscles of the soft palate [13]. Our findings implicate both autocrine and paracrine signaling in the soft palate region, since expression of Wnt, Hh, and Fgf signaling was present in epithelial, CNC-derived and myogenic cells, implying that these signaling pathways may regulate early soft palate development through epithelial-to-mesenchymal and mesenchymal-to-myogenic cell-cell interactions.

Precise understanding of the signaling pathways involved in the development of the soft palate is necessary to understand signaling instruction for the formation and patterning of the soft palate muscles, which is critical for the repair and regeneration of myogenic tissues. The pathway analysis presented in this study provides an important foundation for better understanding of the development of the soft palate myogenic cells and their close interaction with CNC-derived cells. Future studies using tissue-specific conditional knockout mice are necessary to analyze in detail the function of the suggested signaling network in this region. The integration of knowledge gained from expression patterns and conditional knockout mouse models will elucidate the complex regulatory networks and their specific functions controlling palate development.

## Supporting information

S1 Fig Active β-catenin is expressed throughout the embryonic development of the murine soft palate. (A-L) Immunofluorescence for active β-catenin and MHC at E14.5 (A-D), E16.5 (E-H) and E18.5 (I-L). Schematic drawings of the mouse head in the top panel depict the position and angle of the sections. LVP, levator veli palatini; MHC, myosin heavy chain; PLG, palatoglossus; PLP, palatopharyngeus; TVP, tensor veli palatini. Scale bar (A-L) = 50 μm.
(TIF)

## References

[1] Lieberman DE. The evolution of the human head. 1st ed Cambridge (MA): Belknap Press of Harvard University Press; 2011.

[2] Tarr JT, Lambi AG, Bradley JP, Barbe MF, Popoff SN. Development of normal and cleft palate: A central role for connective tissue growth factor (CTGF)/CCN2. J Dev Biol. 2018;6(3):23. 10.3390/jdb6030018 PMC6162467 30029495

[3] Danescu A, Mattson M, Dool C, Diewert VM, Richman JM. Analysis of human soft palate morphogenesis supports regional regulation of palatal fusion. J Anat. 2015;227(4):474–86. 10.1111/joa.12365 26299693 PMC4580105

[4] Chai Y, Maxson RE. Recent advances in craniofacial morphogenesis. Dev Dyn. 2006;235(9):2353–75. 10.1002/dvdy.20833 16680722

[5] Evans A, Ackermann B, Driscoll T. Functional anatomy of the soft palate applied to wind playing. Med Probl Perform Art. 2010;25(4):183–9. 21170481

[6] Wehby GL, Cassell CH. The impact of orofacial clefts on quality of life and healthcare use and costs. Oral Dis. 2010;16(1):3–10. 10.1111/j.1601-0825.2009.01588.x 19656316 PMC2905869

[7] Hunt O, Burden D, Hepper P, Johnston C. The psychosocial effects of cleft lip and palate: a systematic review. Eur J Orthod. 2005;27(3):274–85. 10.1093/ejo/cji004 15947228

[8] Monroy PLC, Grefte S, Kuijpers-Jagtman AM, Wagener FADTG, Von den Hoff JW. Strategies to improve regeneration of the soft palate muscles after cleft palate repair. Tissue Eng Part B Rev. 2012;18(6):468–77. 10.1089/ten.TEB.2012.0049 22697475 PMC3696944

[9] Li J, Rodriguez G, Han X, Janeckova E, Kahng S, Song B, et al Regulatory mechanisms of soft palate development and malformations. J Dent Res. 2019;98(9):959–67. 10.1177/0022034519851786 31150594 PMC6651766

[10] Grimaldi A, Parada C, Chai Y. A comprehensive study of soft palate development in mice. Plos One. 2015;10(12):15. 10.1371/journal.pone.0145018 PMC4687642 26671681

[11] Cordero DR, Brugmann S, Chu YN, Bajpai R, Jame M, Helms JA. Cranial neural crest cells on the move: their roles in craniofacial development. Am J Med Genet A. 2011;155A(2):270–9. 10.1002/ajmg.a.33702 21271641 PMC3039913

[12] Iwata J, Suzuki A, Yokota T, Ho TV, Pelikan R, Urata M, et al TGFβ regulates epithelial-mesenchymal interactions through WNT signaling activity to control muscle development in the soft palate. Development. 2014;141(4):909–17. 10.1242/dev.103093 24496627 PMC3912833

[13] Sugii H, Grimaldi A, Li JY, Parada C, Thach VH, Feng JF, et al The Dlx5-FGF10 signaling cascade controls cranial neural crest and myoblast interaction during oropharyngeal patterning and development. Development. 2017;144(21):4037–45. 10.1242/dev.155176 28982687 PMC5702075

[14] Lin CX, Fisher AV, Yin Y, Maruyama T, Veith GM, Dhandha M, et al The inductive role of Wnt-β-Catenin signaling in the formation of oral apparatus. Dev Biol. 2011;356(1):40–50. 10.1016/j.ydbio.2011.05.002 21600200 PMC3130801

[15] Huelsken J, Vogel R, Brinkmann V, Erdmann B, Birchmeier C, Birchmeier W. Requirement for β-catenin in anterior-posterior axis formation in mice. J Cell Biol. 2000;148(3):567–78. 10.1083/jcb.148.3.567 10662781 PMC2174807

[16] Brault V, Moore R, Kutsch S, Ishibashi M, Rowitch DH, McMahon AP, et al Inactivation of the (β)-catenin gene by Wnt1-Cre-mediated deletion results in dramatic brain malformation and failure of craniofacial development. Development. 2001;128(8):1253–64. 11262227 10.1242/dev.128.8.1253

[17] Reid BS, Yang H, Melvin VS, Taketo MM, Williams T. Ectodermal Wnt/β-catenin signaling shapes the mouse face. Dev Biol. 2011;349(2):261–9. 10.1016/j.ydbio.2010.11.012 21087601 PMC3057077

[18] Wang YP, Song LY, Zhou CJJ. The canonical Wnt/β-catenin signaling pathway regulates Fgf signaling for early facial development. Dev Biol. 2011;349(2):250–60. 10.1016/j.ydbio.2010.11.004 21070765

[19] Chen JQ, Lan Y, Baek JA, Gao Y, Jiang RL. Wnt/beta-catenin signaling plays an essential role in activation of odontogenic mesenchyme during early tooth development. Dev Biol. 2009;334(1):174–85. 10.1016/j.ydbio.2009.07.015 19631205 PMC2752344

[20] Chiquet BT, Blanton SH, Burt A, Ma D, Stal S, Mulliken JB, et al Variation in WNT genes is associated with non-syndromic cleft lip with or without cleft palate. Hum Mol Genet. 2008;17(14):2212–8. 10.1093/hmg/ddn121 18413325 PMC2852032

[21] Fontoura C, Silva RM, Granjeiro JM, Letra A. Association of WNT9B gene polymorphisms with nonsyndromic cleft lip with or without cleft palate in Brazilian nuclear families. Cleft Palate Craniofac J. 2015;52(1):44–8. 10.1597/13-146 24437584 PMC4102668

[22] Lan Y, Ryan RC, Zhang ZY, Bullard SA, Bush JO, Maltby KM, et al Expression of Wnt9b and activation of canonical Wnt signaling during midfacial morphogenesis in mice. Dev Dyn. 2006;235(5):1448–54. 10.1002/dvdy.20723 16496313 PMC2559872

[23] Suzuki A, Minamide R, Iwata J. WNT/β-catenin signaling plays a crucial role in myoblast fusion through regulation of nephrin expression during development. Development. 2018;145(23):8. 10.1242/dev.168351 PMC6288386 30389854

[24] Zhong Z, Zhao H, Mayo J, Chai Y. Different requirements for Wnt signaling in tongue myogenic subpopulations. J Dent Res. 2015;94(3):421–9. 10.1177/0022034514566030 25576472 PMC4336158

[25] Hutcheson DA, Zhao J, Merrell A, Haldar M, Kardon G. Embryonic and fetal limb myogenic cells are derived from developmentally distinct progenitors and have different requirements for β-catenin. Genes Dev. 2009;23(8):997–1013. 10.1101/gad.1769009 19346403 PMC2675868

[26] Zhang XM, Ramalho-Santos M, McMahon AP. Smoothened mutants reveal redundant roles for Shh and Ihh signaling including regulation of L/R symmetry by the mouse node. Cell. 2001;106(2):781–92. 11517919

[27] Jeong JH, Mao JH, Tenzen T, Kottmann AH, McMahon AP. Hedgehog signaling in the neural crest cells regulates the patterning and growth of facial primordia. Genes Dev. 2004;18(8):937–51. 10.1101/gad.1190304 15107405 PMC395852

[28] Lan Y, Jiang R. Sonic hedgehog signaling regulates reciprocal epithelial-mesenchymal interactions controlling palatal outgrowth. Development. 2009;136(8):1387–96. 10.1242/dev.028167 19304890 PMC2687468

[29] Hammond NL, Brookes KJ, Dixon MJ. Ectopic Hedgehog signaling causes cleft palate and defective osteogenesis. J Dent Res. 2018;97(13):1485–93. 10.1177/0022034518785336 29975848 PMC6262265

[30] Han J, Mayo J, Xu X, Li JY, Bringas P, Maas RL, et al Indirect modulation of Shh signaling by Dlx5 affects the oral-nasal patterning of palate and rescues cleft palate in Msx1-null mice. Development. 2009;136(24):4225–33. 10.1242/dev.036723 19934017 PMC2781056

[31] Rice R, Spencer-Dene B, Connor EC, Gritli-Linde A, McMahon AP, Dickson C, et al Disruption of Fgf10/Fgfr2b-coordinated epithelial-mesenchymal interactions causes cleft palate. J Clin Invest. 2004;113(12):1692–700. 10.1172/JCI20384 15199404 PMC420504

[32] Sagai T, Amano T, Tamura M, Mizushina Y, Sumiyama K, Shiroishi T. A cluster of three long-range enhancers directs regional Shh expression in the epithelial linings. Development. 2009;136(10):1665–74. 10.1242/dev.032714 19369396

[33] Rice R, Connor E, Rice DPC. Expression patterns of Hedgehog signalling pathway members during mouse palate development. Gene Expr Patterns. 2006;6(2):206–12. 10.1016/j.modgep.2005.06.005 16168717

[34] Riley BM, Mansilla MA, Ma J, Daack-Hirsch S, Maher BS, Raffensperger LM, et al Impaired FGF signaling contributes to cleft lip and palate. Proc Nat Acad Sci U.S.A. 2007;104(11):4512–7. 10.1073/pnas.0607956104 PMC1810508 17360555

[35] De Moerlooze L, Spencer-Dene B, Revest JM, Hajihosseini M, Rosewell I, Dickson C. An important role for the IIIb isoform of fibroblast growth factor receptor 2 (FGFR2) in mesenchymal-epithelial signalling during mouse organogenesis. Development. 2000;127(3):483–92. 10631169 10.1242/dev.127.3.483

[36] Wang C, Chang JYF, Yang CF, Huang YQ, Liu JC, You P, et al Type 1 Fibroblast growth factor receptor in cranial neural crest cell-derived mesenchyme is required for palatogenesis. J Biol Chem. 2013;288(30):22174–83. 10.1074/jbc.M113.463620 23754280 PMC3724669

[37] Yu K, Karuppaiah K, Ornitz DM. Mesenchymal fibroblast growth factor receptor signaling regulates palatal shelf elevation during secondary palate formation. Dev Dyn. 2015;244(11):1427–38. 10.1002/dvdy.24319 26250517 PMC4619180

[38] Jin JZ, Lei ZM, Lan ZJ, Mukhopadhyay P, Ding JX. Inactivation of Fgfr2 gene in mouse secondary palate mesenchymal cells leads to cleft palate. Reprod Toxicol. 2018;77:137–42. 10.1016/j.reprotox.2018.03.004 29526646

[39] Weng MJ, Chen ZX, Xiao Q, Li RM, Chen ZQ. A review of FGF signaling in palate development. Biomed Pharmacother. 2018;103:240–7. 10.1016/j.biopha.2018.04.026 29655165

[40] Olwin BB, Arthur K, Hannon K, Hein P, McFall A, Riley B, et al Role of Fgfs in skeletal muscle and limb development. Mol Reprod Dev. 1994;39(1):90–101. 10.1002/mrd.1080390114 7999366

[41] Ciruna B, Rossant J. Fgf signaling regulates mesoderm cell fate specification and morphogenetic movement at the primitive streak. Dev Cell. 2001;1(1):37–49. 11703922 10.1016/s1534-5807(01)00017-x

[42] Smith TM, Lozanoff S, Iyyanar PP, Nazarali AJ. Molecular signaling along the anterior-posterior axis of early palate development. Front Physiol. 2013;3:14. 10.3389/fphys.2012.00488 PMC3539680 23316168

[43] Reynolds K, Kumari P, Rincon LS, Gu R, Ji Y, Kumar S, et al Wnt signaling in orofacial clefts: crosstalk, pathogenesis and models. Dis Models Mech. 2019;12(2):24. 10.1242/dmm.037051 PMC6398499 30760477

[44] Lee JM, Kim JY, Cho KW, Lee MJ, Cho SW, Kwak S, et al Wnt11/Fgfr1b cross-talk modulates the fate of cells in palate development. Dev Biol. 2008;314(2):341–50. 10.1016/j.ydbio.2007.11.033 18191119

[45] Bai CB, Auerbach W, Lee JS, Stephen D, Joyner AL. Gli2, but not Gli1, is required for initial Shh signaling and ectopic activation of the Shh pathway. Development. 2002;129(20):4753–61. 12361967 10.1242/dev.129.20.4753

[46] Lustig B, Jerchow B, Sachs M, Weiler S, Pietsch T, Karsten U, et al Negative feedback loop of Wnt signaling through upregulation of conductin/Axin2 in colorectal and liver tumors. Mol Cell Biol. 2002;22(4):1184–93. 10.1128/MCB.22.4.1184-1193.2002 11809809 PMC134640

[47] Menezes R, Letra A, Kim AH, Kuchler EC, Day A, Tannure PN, et al Studies with Wnt genes and nonsyndromic cleft lip and palate. Birth Defects Res A Clin Mol Teratol. 2010;88(11):995–1000. 10.1002/bdra.20720 20890934 PMC2991560

[48] He FL, Xiong W, Wang Y, Li L, Liu C, Yamagami T, et al Epithelial Wnt/β-catenin signaling regulates palatal shelf fusion through regulation of Tgfβ3 expression. Dev Biol. 2011;350(2):511–9. 10.1016/j.ydbio.2010.12.021 21185284 PMC3040240

[49] Hagemann AIH, Scholpp S. The tale of the three brothers—Shh, Wnt, and Fgf during development of the thalamus. Front Neurosci. 2012;6:9. 22654733 10.3389/fnins.2012.00076 PMC3361129

[50] Yang YZ, Kozin SH. Cell signaling regulation of vertebrate limb growth and patterning. J Bone Joint Surg Am. 2009;91A:76–80. 10.2106/JBJS.I.00079 PMC2698794 19571072

[51] Aman AJ, Fulbright AN, Parichy DM. Wnt/β-catenin regulates an ancient signaling network during zebrafish scale development. Elife. 2018;7:22. 10.7554/eLife.37001 PMC6072442 30014845

[52] Tucker A, Sharpe P. The cutting-edge of mammalian development; How the embryo makes teeth. Nat Rev Genet. 2004;5(7):499–508. 10.1038/nrg1380 15211352

[53] Liu F, Wang SL. Molecular cues for development and regeneration of salivary glands. Histol Histopathol. 2014;29(3):305–12. 10.14670/HH-29.305 24189993 PMC4623581

[54] Tucker AS. Salivary gland development. Semin Cell Dev Biol. 2007;18(2):237–44 10.1016/j.semcdb.2007.01.006 17336109

[55] Volckaert T, De Langhe SP. Wnt and FGF mediated epithelial-mesenchymal crosstalk during lung development. Dev Dyn. 2015;244(3):342–66. 10.1002/dvdy.24234 25470458 PMC4344844

[56] Warner DR, Smith HS, Webb CL, Greene RM, Pisano MM. Expression of Wnts in the developing murine secondary palate. Int J Dev Biol. 2009;53(7):1105–12. 10.1387/ijdb.082578dw 19598129 PMC2746657

[57] Jin YR, Han XH, Taketo MM, Yoon JK. Wnt9b-dependent FGF signaling is crucial for outgrowth of the nasal and maxillary processes during upper jaw and lip development. Development. 2012;139(10):1821–30. 10.1242/dev.075796 22461561 PMC3328181

[58] Alappat SR, Zhang ZY, Suzuki K, Zhang XY, Liu HB, Jiang RL, et al The cellular and molecular etiology of the cleft secondary palate in Fgf10 mutant mice. Dev Biol. 2005;277(1):102–13. 10.1016/j.ydbio.2004.09.010 15572143

[59] Teshima THN, Lourenco SV, Tucker AS. Multiple cranial organ defects after conditionally knocking out Fgf10 in the neural crest. Front Physiol. 2016;7:10. 27826253 10.3389/fphys.2016.00488 PMC5078472

[60] Puthiyaveetil JSV, Kota K, Chakkarayan R, Chakkarayan J, Thodiyil AKP. Epithelial—mesenchymal interactions in tooth development and the significant role of growth factors and genes with emphasis on mesenchyme—A review. J Clin Diagn Res. 2016;10(9):ZE5–ZE9. 10.7860/JCDR/2016/21719.8502 PMC5072096 27790596

[61] Ribatti D, Santoiemma M. Epithelial-mesenchymal interactions: a fundamental developmental biology mechanism. Int J Dev Biol. 2014;58(5):303–6. 10.1387/ijdb.140143dr 25354449

[62] Cunha GR, Hom YK. Role of Mesenchymal-epithelial interactions in mammary gland development. J Mammary Gland Biol Neoplasia. 1996;1(1):21–35. 10887478 10.1007/BF02096300

[63] Iwata J, Parada C, Chai Y. The mechanism of TGF-β signaling during palate development. Oral Dis. 2011;17(8):733–44. 21395922 10.1111/j.1601-0825.2011.01806.x PMC3329177

[64] Deb A. Cell cell interaction in the heart via Wnt/β-catenin pathway after cardiac injury. Cardiovasc Res. 2014;102(2):214–23. 10.1093/cvr/cvu054 24591151 PMC3989450

[65] Hosokawa R, Oka K, Yamaza T, Iwata J, Urata M, Xu X, et al TGF-beta mediated Fgf10 signaling in cranial neural crest cells controls development of myogenic progenitor cells through tissue-tissue interactions during tongue morphogenesis. Dev Biol. 2010;341(1):186–95. 10.1016/j.ydbio.2010.02.030 20193675 PMC3336866

