# Supplementary figures and images for: Dynamic activation of Wnt, Fgf, and Hh signaling during soft palate development
Source: PLoS One. 2019 Oct 15;14(10):e0223879. doi: 10.1371/journal.pone.0223879 (PMC6793855; doi:10.1371/journal.pone.0223879)

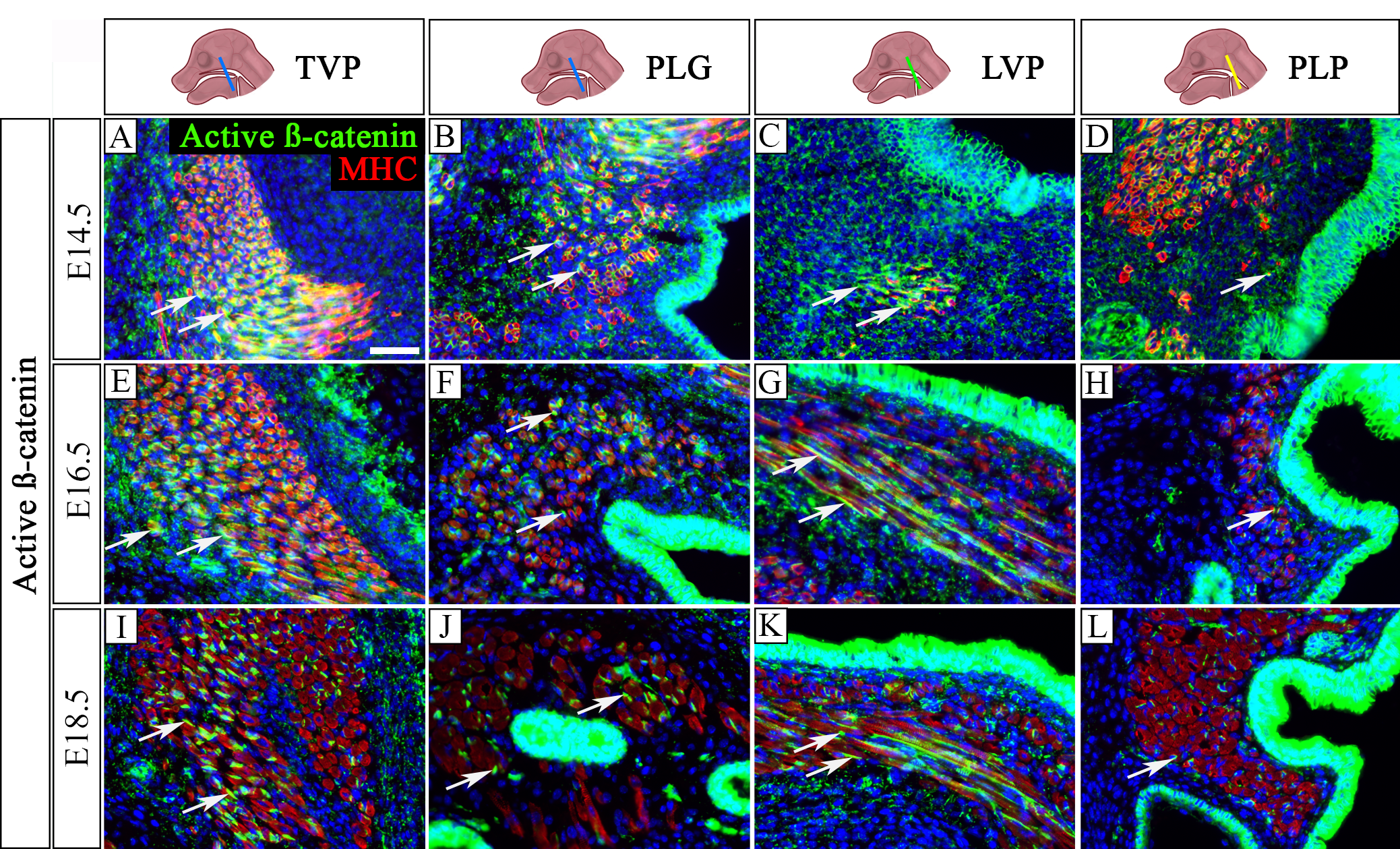

Supplement: S1 Fig — (A-L) Immunofluorescence for active β-catenin and MHC at E14.5 (A-D), E16.5 (E-H) and E18.5 (I-L). Schematic drawings of the mouse head in the top panel depict the position and angle of the sections. LVP, levator veli palatini; MHC, myosin heavy chain; PLG, palatoglossus; PLP, palatopharyngeus; TVP, tensor veli palatini. Scale bar (A-L) = 50 μm. (TIF) [file pone.0223879.s001.tif]
